# Inhibitory Effects of Peptide Lunasin in Colorectal Cancer HCT-116 Cells and Their Tumorsphere-Derived Subpopulation

**DOI:** 10.3390/ijms21020537

**Published:** 2020-01-14

**Authors:** Samuel Fernández-Tomé, Fei Xu, Yanhui Han, Blanca Hernández-Ledesma, Hang Xiao

**Affiliations:** 1 Instituto de Investigación en Ciencias de la Alimentación (CIAL, CSIC-UAM CEI UAM+CSIC), Nicolás Cabrera, 9, 28049 Madrid, Spain; fernandeztome.samuel@gmail.com; 2 Department of Food Science, University of Massachusetts, Amherst, MA 01003, USA; xufei5056@gmail.com (F.X.); yanhuihan@foodsci.umass.edu (Y.H.)

**Keywords:** colorectal cancer, cancer stem cells, chemoprevention, bioactive peptide, lunasin

## Abstract

The involvement of cancer stem-like cells (CSC) in the tumor pathogenesis has profound implications for cancer therapy and chemoprevention. Lunasin is a bioactive peptide from soybean and other vegetal sources with proven protective activities against cancer and other chronic diseases. The present study focused on the cytotoxic effect of peptide lunasin in colorectal cancer HCT-116 cells, both the bulk tumor and the CSC subpopulations. Lunasin inhibited the proliferation and the tumorsphere-forming capacity of HCT-116 cells. Flow cytometry results demonstrated that the inhibitory effects were related to apoptosis induction and cell cycle-arrest at G1 phase. Moreover, lunasin caused an increase in the sub-GO/G1 phase of bulk tumor cells, linked to the apoptotic events found. Immunoblotting analysis further showed that lunasin induced apoptosis through activation of caspase-3 and cleavage of PARP, and could modulate cell cycle progress through the cyclin-dependent kinase inhibitor p21. Together, these results provide new evidence on the chemopreventive activity of peptide lunasin on colorectal cancer by modulating both the parental and the tumorsphere-derived subsets of HCT-116 cells.

## 1. Introduction

Current statistics on colorectal cancer (CRC) have ranked this disease as the third most commonly diagnosed malignancy and the fourth leading cause of cancer death in the world [1]. In recent years, a great deal of research has been focused on CRC pathogenesis. Meanwhile, the existence of tumor-initiating cells or cancer stem-like cells (CSC) in this solid tumor has been established [2,3,4]. According to the CSC theory, a minor population of tumor cells is responsible for the driving of tumorigenesis [5]. These stem cells, like those in adult tissue, undergo unlimited proliferation and asymmetrically division into more differentiated cells leading to the neoplastic growth and maintenance [6]. In addition, it has been suggested that this CSC subpopulation might be potentially responsible for the tumor invasion, metastasis, recurrence, and resistance to therapy [7,8]. Therefore, the potential of preventive strategies needs to be evaluated not only against CRC cells, representing the bulk of the tumor mass (non-CSC), but also against colon CSC.

Accumulating evidence and epidemiological studies have revealed an inverse correlation between soybean consumption and the risk of CRC development [9,10], that can be in part due to the chemopreventive effects of the bioactive compounds described in this legume. Several soybean components including isoflavones [11], saponins [12], and bioactive proteins and peptides, such as lectins and protease inhibitors [13,14] have been shown to exert protective activities against the growth of CRC cells. Identified in soybean, lunasin is a bioactive peptide which chemopreventive properties have been recently reviewed [15]. It has been demonstrated that lunasin is able to cause cytotoxicity in four different human CRC cell lines, HCT-116, HT-29, KM12L4, and RKO, and their oxaliplatin-resistant variants [16]. Studies on the mechanisms of action involved in this antiproliferative activity have been mostly carried out in HT-29 and KM12L4 cells, in which Dia and de Mejia demonstrated lunasin´s effects on apoptosis-induction, cell cycle progression, and modulation of CRC-related biomarkers [16,17,18]. Moreover, García-Nebot and others reported the protective role played by lunasin in differentiated Caco-2 cells, as a model of human enterocytes, exposed to oxidizing agents through promoting cell viability and counteracting the rise in reactive oxygen species levels [19]. This notably antioxidant protection at intestinal level is also a noteworthy aspect, pointing lunasin as a promising chemopreventive agent against CRC.

The emergence of the CSC model has profound implications on cancer chemoprevention and the search of natural components targeting these cells has been markedly prompted [20]. Some dietary compounds and phytochemicals have been shown to potentially interact toward the pathways involved in the renewal and proliferation of CSC [21,22,23]. Despite the fact that food proteins and peptides have received increasing attention for their efficacy preventing the different stages of cancer, including initiation, promotion, and progression [24,25], their protective role against CSC has been scarcely studied. Accordingly, this study aimed to evaluate the cytotoxicity of peptide lunasin in human CRC HCT-116 cells by evaluating its inhibitory capacity on cell viability and CSC-related tumorsphere forming activity, as well as its effects on apoptosis induction, cell cycle progression, and carcinogenesis-related protein biomarkers.

## 2. Results and Discussion

### 2.1. Inhibitory Effect of Lunasin on Cell Viability and Tumorsphere Formation 

**Figure 1:**
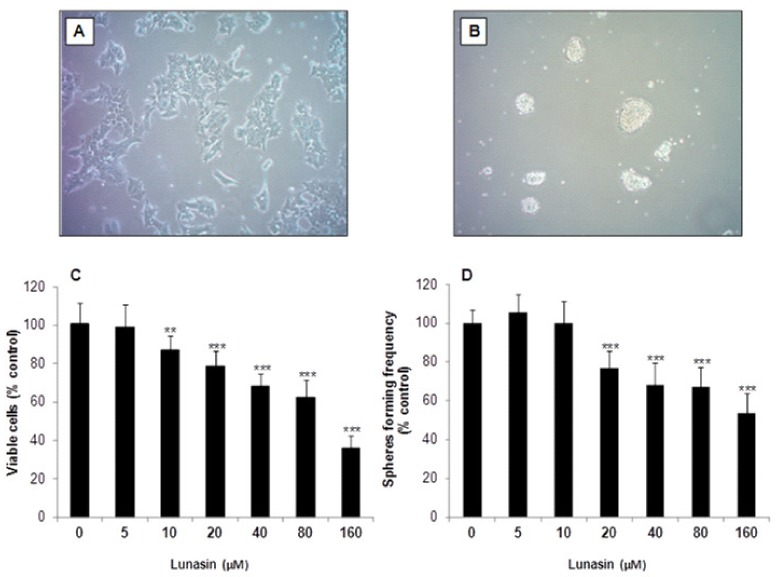
Cell culture and cytotoxic effect of lunasin on colorectal cancer (CRC) cells. Representative images of (**A**) HCT-116 cells in adherent conditions and (**B**) enrichment culture of tumor-derived colon-spheres formed from the parental HCT-116 cell line under anchorage-independent conditions. (**C**) HCT-116 cells were treated with lunasin (5–160 μM) for 72 h, and cell viability was determined by the MTT assay. (**D**) Colon tumorspheres were treated with lunasin (5–160 μM) for 10 days, stained with crystal violet solution and counted. Results, expressed as percentage of control cells, are means ± standard deviation (SD) of the replicates of experiments carried out. ** (*p* < 0.01), *** (*p* < 0.001) significantly different from control.

In this study, the human HCT-116 cell line was grown in monolayer as parental CRC cells (Figure 1A) and used for the enrichment of tumor-derived colon-spheres (Figure 1B). We first examined the growth of adherent HCT-116 cells exposed to lunasin. HCT-116 cells were treated with serial concentrations of synthetic lunasin (5–160 μM) for 72 h and the number of viable cells was assessed by the 3-(4,5-dimethylthiazol-2-yl)-2,5-diphenyl tetrazolium bromide (MTT) assay. As shown in Figure 1C, lunasin showed cell proliferation inhibitory properties with increasing effects at higher doses. Hence, treatment with 10 µM lunasin was able to induce a significant reduction on cellular growth (12.9%, *p* < 0.01) compared to control cells. The cytotoxic effect increased up to the highest concentration used (64.1%, *p* < 0.001). The IC_50_ value, expressed as the peptide concentration needed to inhibit 50% of cell number, was 107.5 ± 1.9 µM. It had been previously demonstrated that lunasin is able to induce cytotoxicity in colon cancer HCT-116, HT-29, KM12L4, and RKO cells, with IC_50_ values of 26.3, 61.7, 13.0, and 21.6 µM, respectively, while it was no toxic for colon fibroblasts CCD-33Co [16]. While these authors used purified lunasin (~90%) from defatted soybean flour, in our study we have assessed the effects of synthetic lunasin. The higher IC_50_ value found in our study might be due to differences in the secondary and tertiary structures between plant-purified lunasin and the synthetic peptide. Additionally, other compounds present in the natural preparation could be responsible for the observed change in the inhibitory potency. In this regard, synthetic lunasin has been shown to suppress the growth of breast cancer MDA-MB-231 cells with a reported IC_50_ value of 181.0 µM [26]. 

Since colon-sphere subpopulations were demonstrated to exert a key role in the CRC pathogenesis, the culture of tumor-derived spheroids has been widely used for the evaluation of chemotherapy drugs and chemopreventive agents [3]. The sphere formation assay is extensively applied as in vitro method for the derivation and characterization of stem-like cancer cells with intrinsic self-renewal and tumorigenic properties [27]. To evaluate whether lunasin might prevent the formation of CRC-derived colon-spheres, we performed the colony formation assay as we did previously [28], following some modifications to model the enrichment of tumor-derived spheroids in culture. Hence, colon-tumorspheres were enriched from adherent HCT-116 cells, cultured as non-adherent spheres under anchorage-independent conditions, and treated with lunasin for 10 days (Figure 1B). As shown in Figure 1D, lunasin at the lowest range assessed (5–10 µM) was not able to suppress tumorsphere-forming capacity. Likewise, Pabona and others had reported that while isoflavone genistein (40 nM) reduced the number of mammosphere-forming units in malignant breast cancer MCF-7 cells, peptide lunasin (2 µM) was not able to recapitulate this inhibitory protection [29]. However, as represented in Figure 1D, the peptide in the range of 20–160 µM, exerted a significant inhibitory effect (*p* < 0.001). Evidence supports that colon-spheres formed by culture in ultra-low attachment conditions in supplemented-serum-free medium presented more stem-like cell properties [30]. Following this culture, spheroid formation of DLD-1 and SW480 CRC cells with protein and mRNA expression of CSC markers including CD133, CD44, ALDHA1, Oct-4 and Nanog, was recently inhibited by (−)-epigallocatechin-3-gallate [31]. However, the characterization of these markers was not performed in the present study. The calculated IC_50_ value for HCT-116-derived spheres in our study was 161.0 ± 2.4 µM, indicating that colony-forming cells are less sensitive to peptide lunasin than parental HCT-116 cells (107.5 ± 1.9 μM). These results are in agreement with the reported higher resistance of CSC to other anti-cancer therapies [7,32]. Similarly, Yang and others have shown that docosahexaenoic acid (DHA) exerts higher antiproliferative potency on adherent CRC SW620 cells than on their tumorspheres-derived CSC subpopulation [33]. Nevertheless, in the study of McConnell and others, it was found that peptide lunasin presented a higher anti-proliferative activity against non-small cell lung cancer cells when they were assessed under anchorage-independent growth conditions, compared to anchorage-dependent conditions [34]. In this line, detailed studies on soybean lunasin effects against melanoma CSC have been recently published [35,36]. These authors found that lunasin specifically targeted the cancer-initiating subset of melanoma cancer cells, suppressing not only their oncosphere formation capacity but also the expression of the CSC-markers aldehyde dehydrogenase and Nanog, while also inducing the expression of melanocyte-associated differentiation markers tyrosinase and microphthalmia-associated transcription factor. Interestingly, the functional domain arginine-glycine-aspartic acid (RGD) of lunasin sequence was found to be crucial in the interaction with integrins, cell internalization, inhibition of histone acetylation and anticancer-stem activity [36]. Therefore, lunasin´s modulatory chemoprevention might notably depend on the lunasin´s preparation and origin, as well as on the culture conditions and the cell line used. Inhibitory effects of lunasin over colon-spheres derived from other CRC cell lines apart from HCT-116 cells might be different and thus should be evaluated in future studies with different types of CRC.

### 2.2. Apoptosis Analysis of Lunasin-Treated CRC Cells 

Tumor cell populations expand in number through several molecular processes such as the capability of evading programmed cell death by presenting an elevated apoptotic threshold [37]. In order to determine whether the inhibitory effect of lunasin on HCT-116 cells was through interacting with the apoptotic pathways, adherent and colon-spheres-derived cells were incubated with lunasin, and apoptosis detection was assessed by flow cytometry-based Annexin V/propidium iodide (PI) assay. Annexin V has high affinity for membrane phospholipid phosphatidylserine translocated to the outer cellular environment as one of the earliest processes during apoptosis. Phospholipid phosphatidylserine is exposed before the loss of membrane integrity, which can be revealed in later stages of cell apoptosis or necrosis by the viability dye PI. Based on the lunasin´s inhibitory effects on HCT-116 cell viability and colon-sphere forming-frequency, the range of 20–80 µM for this peptide was then chosen as the optimal treatment concentration for subsequent experiments. 

**Figure 2:**
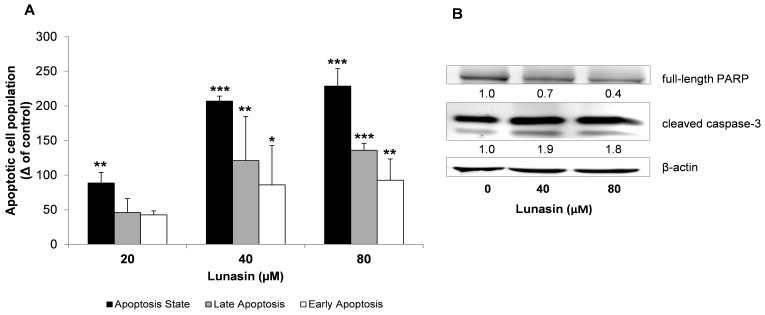
Effect of lunasin on the apoptosis state of HCT-116 cells. Cells were treated with lunasin at the indicated concentrations (20, 40, and 80 μM) for 72 h, and harvested for apoptosis analysis and Western immunoblotting. (**A**) Flow cytometry-based Annexin V/PI double labeling of apoptotic cells. Total apoptotic cells were identified as Annexin V-positive cells (apoptosis state), being Annexin V-positive/PI-negative and Annexin V-positive/PI-positive cells identified as early apoptotic and late apoptotic cells, respectively. Results, presented as the increased number in apoptotic cell populations compared to control cells, are means ± standard deviation (SD) of the replicates of experiments carried out. * (*p* < 0.05), ** (*p* < 0.01), *** (*p* < 0.001) significantly different from control. (**B**) Expression of full length PARP and cleaved caspase-3 proteins determined by Western Blot. The numbers underneath the blots represent band intensity that was normalized to β-actin and measured by Image J software (means of duplicates, and standard deviations within ± 15% of the means were not shown). β-actin was served as an equal loading control for cytosolic fraction.

Figure 2 presents the apoptotic state of adherent HCT-116 cells under control and lunasin-treated conditions for 72 h. The apoptotic populations of cells treated with the peptide were significantly increased (Figure 2A). Lunasin at 20, 40 and 80 µM induced 1.3, 1.7 and 1.8-fold increase of total apoptotic cells, respectively, compared to control. In the case of lunasin at 40 and 80 µM, cells both in the early and late apoptotic stages were significantly enhanced. The apoptosis-involved inhibitory role of lunasin against HCT-116 cells was further addressed by the immunoblotting study of the molecular proteins PARP and caspase-3. PARP is responsible for the regulation of many cellular functions, such as key events supporting cell viability and DNA repair [38]. PARP degradation has been shown to facilitate cellular disassembly, and serve as a marker of cells undergoing apoptosis, with this protein being the main cleavage target on the activity of the apoptotic trigger caspase-3 [39]. As shown in Figure 2B, lunasin activated the cleavage of caspase-3 and, consequently, the protein level of full-length PARP was decreased in lunasin-treated cells. This might be accompanied to increased expression of cleaved PARP, a hallmark of apoptosis, as we found previously [40]. In this line, Dia and de Mejia found that lunasin was able to activate the apoptotic mitochondrial pathway in HT-29 and KM12L4 cells, as evidenced by the modulation of Bcl-2/Bax family of proteins, nuclear clusterin, cytochrome c, and caspases-activity [16,17]. Similar apoptosis-related properties have been reported for this peptide against the growth of leukemia L1210 cells [41], and breast cancer MCF-7 and MDA-MB-231 cells [29,42].

**Figure 3:**
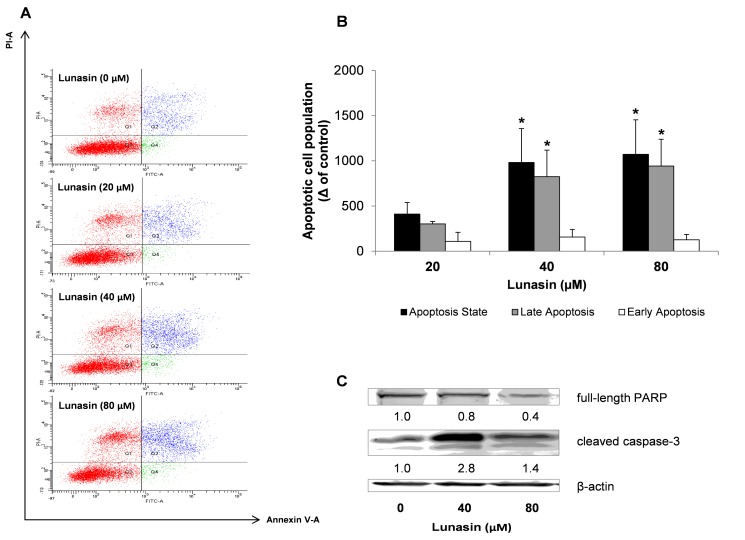
Effect of lunasin on the apoptosis state of colon tumorspheres. Cells were treated with lunasin at the indicated concentrations for 7 days, and harvested for apoptosis analyses and Western immunoblotting. (**A**) Flow cytometry-based Annexin V/PI double labeling of apoptotic cells. (**B**) Total apoptotic cells were identified as Annexin V-positive cells (apoptosis state), being Annexin V-positive/PI-negative and Annexin V-positive/PI-positive cells identified as early apoptotic and late apoptotic cells, respectively. Results, presented as the increased number in apoptotic cell populations compared to control cells, are means ± standard deviation (SD) of the replicates of experiments carried out. * (*p* < 0.05) significantly different from control. (**C**) Expression of full length PARP and cleaved caspase-3 proteins determined by Western Blot. The numbers underneath the blots represent band intensity that was normalized to β-actin and measured by Image J software (means of duplicates, and standard deviations within ± 15% of the means were not shown). β-actin was served as an equal loading control for cytosolic fraction.

We next aimed to determine whether the apoptosis-inducing property was also involved in the suppression of the spheroid-forming capacity of HCT-116 cells. Colon-spheres were treated with lunasin for 7 days and apoptosis detection was examined as shown in Figure 3. Results from the flow cytometry study after staining with Annexin-V/PI showed that lunasin led to induction in the cellular apoptotic state (Figure 3A,B). The raise in the number of apoptotic cells was not significantly promoted at lunasin 20 µM. However, lunasin both at 40 and 80 µM exerted a 2.0-fold apoptosis-induction effect, mostly in the late apoptotic cellular subset, independently of the dose. As shown in Figure 3C, the implication of the mechanism responsible for the inhibitory effect of lunasin peptide against the expansion of the HCT-116-derived colon-spheres was further demonstrated by immunoblotting. Again, cleaved caspase-3 activity was induced by lunasin treatment, with this activation being companied by a decrease of PARP protein levels. Our results suggested a dose-dependent trend in the down-regulated levels of PARP protein after lunasin treatment. However, this was not the case for cleaved caspase-3, which is more related to the inhibitory effects shown in the MTT assay and, mostly, over the spheroid-forming capacity where peptide lunasin displayed a ca. 30–40% inhibitory effect for the dose range of 20–160 µM. Therefore, in the present study, it has been suggested that lunasin has similar effects in the apoptosis-induction of both populations of CRC HCT-116 cells. In this sense, other food/natural compounds and phytochemicals have demonstrated to exert similar inhibitory effects through apoptosis induction against the expansion of the CSC subpopulation not only in CRC [33,43,44], but also in pancreatic and prostate cancer cells [45,46].

### 2.3. Effect of Lunasin on Cell Cycle Progression of CRC Cells

**Figure 4:**
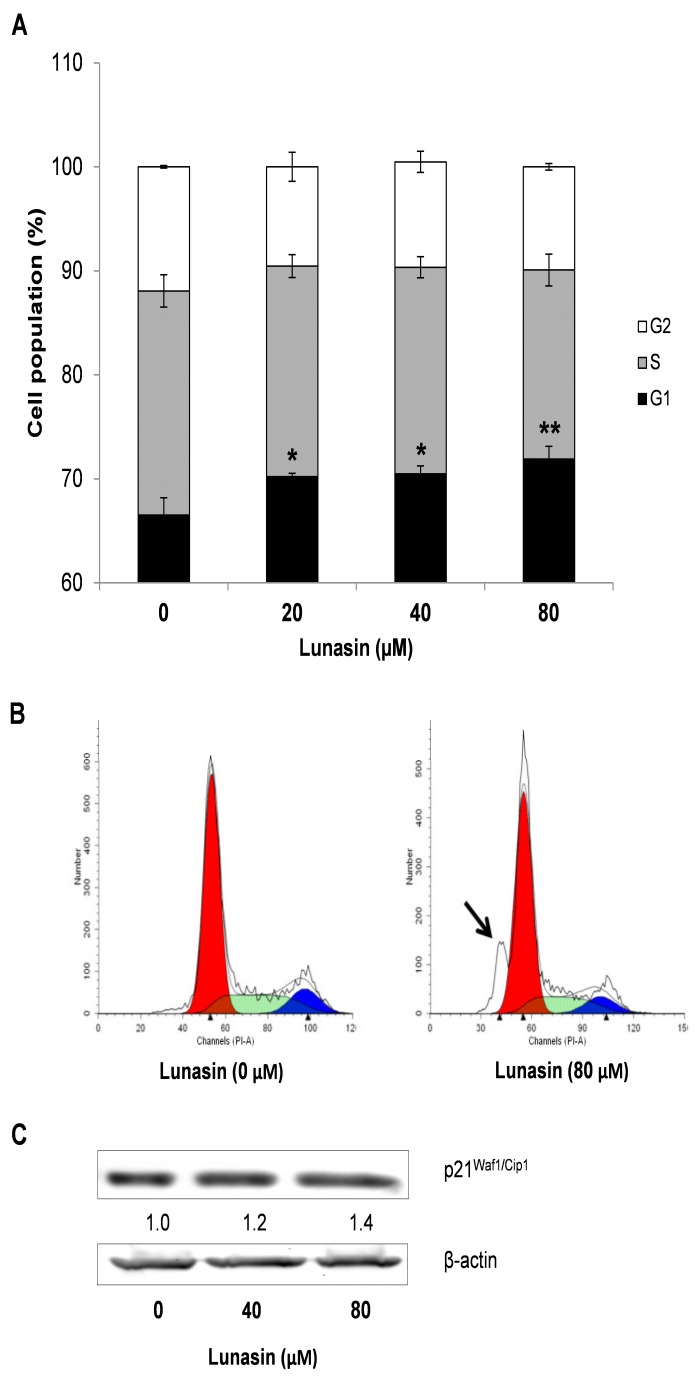
Effect of lunasin on cell cycle progression of HCT-116 cells. Cells were treated with lunasin at the indicated concentrations for 72 h, and harvested for cell cycle analysis and Western immunoblotting. (**A**) Cell cycle distribution was assessed by flow cytometry using PI staining. Results, presented as percentage of cells in G1, S, and G2 phases, are means ± standard deviation (SD) of the replicates of experiments carried out. * (*p* < 0.05), ** (*p* < 0.01) significantly different from control. (**B**) Representative images of lunasin-induced increase in the sub-GO/G1 cell population (black arrow). (**C**) Expression of p21^Waf1/Cip1^ protein determined by Western Blot. The numbers underneath the blots represent band intensity that was normalized to β-actin and measured by Image J software (means of duplicates, and standard deviations within ± 15% of the means were not shown). β-actin was served as an equal loading control for cytosolic fraction.

**Figure 5:**
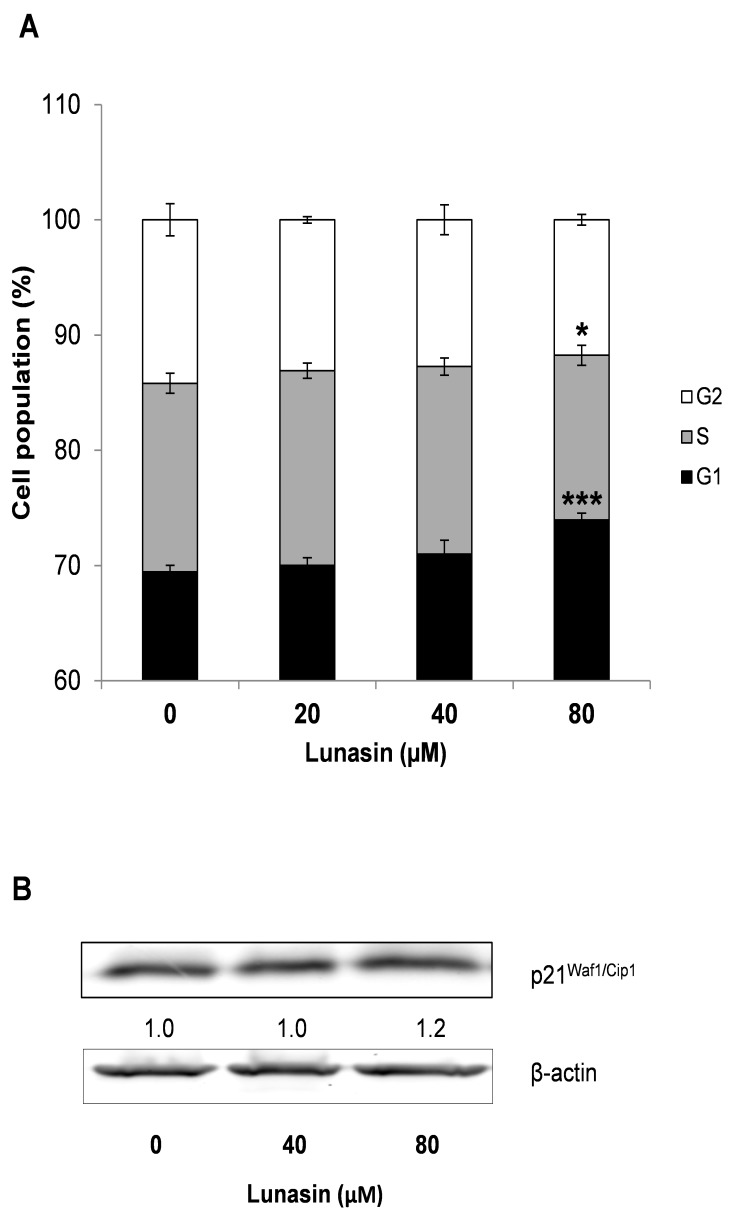
Effect of lunasin on cell cycle progression of colon tumorspheres. Cells were treated with lunasin at the indicated concentrations for 7 days, and harvested for cell cycle analyses and Western immunoblotting. (**A**) Cell cycle distribution was assessed by flow cytometry using PI staining. Results, presented as percentage of cells in G1, S, and G2 phases, are means ± standard deviation (SD) of the replicates of experiments carried out. * (*p* < 0.05), *** (*p* < 0.001) significantly different from control. (**B**) Expression of p21Waf1/Cip1 protein determined by Western Blot. The numbers underneath the blots represent band intensity that was normalized to β-actin and measured by Image J software (means of duplicates, and standard deviations within ± 15% of the means were not shown). β-actin was served as an equal loading control for cytosolic fraction.

To provide further insights into the growth inhibitory effects exerted by lunasin in HCT-116 cells, analyses on cell cycle distribution were performed on both adherent cells and colon-spheres after treatment with lunasin for 72 h and 7 days, respectively. Deregulation of cell cycle control and potential to replicate without limit are one of the hallmarks of cancer, with all these events being highly regulated by internal checkpoints that ensure the proper cellular division [35]. As shown in Figure 4A, control adherent HCT-116 cells were found to significantly increase their G1 phase (66.5 ± 1.7%) after lunasin´s treatment (20 µM lunasin, G1: 70.2 ± 0.3%, *p* < 0.05; 40 µM lunasin, 70.5 ± 0.7%, *P* < 0.05; 80 µM lunasin, 72.0 ± 1.2%, *p* < 0.01). Interestingly, as represented in Figure 4B, lunasin-treated cells also resulted in a marked accumulation of the sub-G0/G1 cell population, compared to control cells. Cells at the sub-G0/G1 fraction contain less amount of DNA than G1 cells, suggesting DNA degradation potentially caused by apoptotic events [47]. This effect had also been demonstrated for peptide lunasin in leukemia L1210 cells [41] and is in agreement with our results on apoptosis-induction in HCT-116 cells (Figure 2). On the other hand, our findings differ with other studies showing the capability of this peptide to arrest cell cycle at S-phase in breast cancer MDA-MB-231 cells [42], and at G2-phase in leukemia L1210 cells [41] and CRC HT-29 and KM12L4 cells [16,17]. However, other RGD-motif-containing peptides have been also reported to result in a G0/G1-phase arrest in cancer cells [48]. Noteworthy, different cancer cells might respond differently to lunasin peptide accordingly to their diverse tumor phenotype. Moreover, regarding to the colon tumorspheres (Figure 5A), lunasin at 80 µM also led to an enhancement of G1-arrest (74.0 ± 0.6%, *p* < 0.001), accompanied with a reduction in the S-cellular subset (14.3 ± 0.9%, *p* < 0.05), compared to control cells (G1: 69.4 ± 0.6%; S: 16.4 ± 0.9%). This effect might be related to the antiproliferative and pro-apoptotic activities above indicated. However, 20–40 µM-treated colon-spheres showed a similar trend but in a weaker manner, lacking statistical significance in this dose range.

To further explain lunasin´s effect on cell cycle progression, evaluation of the expression of the cyclin-dependent kinase (CDK) inhibitors p21^Waf1/Cip1^ and p27^Kip1^ was performed by Western Blot. Treatment of CRC cells with lunasin showed no effect on the level of p27 (data not shown), while it slightly increased the molecular expression of p21 protein up to 140% and 120% in adherent HCT-116 cells (Figure 4C) and colon-spheres CSC (Figure 5B), respectively. A consistent role of lunasin over these molecules cannot be thus extracted from our results. CDK-inhibitors p21 and p27 are two important cell cycle regulators at the G1-phase known to be usually co-regulated, although they have shown paradoxical roles in the literature [49]. In non-small cell lung H661 cancer cells, expressing a mutated form of p53 and thus low non-inducible levels of p21, McConnell and co-workers recently found that peptide lunasin also blocked cell cycle at the G1/S-phase through CDK-inhibitor p27 as well as by disrupted phosphorylation of the retinoblastoma protein [34]. Regarding studies on CRC, Dia and de Mejia reported lunasin´s capability to induce the expression of the CDK-inhibitor p21 in HT-29 and KM12L4 cells, and linked this effect with a decreased cell proliferation, cell cycle arrest, and up-regulation of the pro-apoptotic markers caspase-3 and nuclear clusterin isoform [16,17]. In these studies, CDK-inhibitor p27 induction was also demonstrated in KM12L4 cells [16] although it was not evaluated in the HT-29 cell line [17].

In order to provide more evidence on the cancer-preventive role of bioactive peptide lunasin, specifically against the CRC malignancy, some studies have been carried out. In the highly metastatic KM12L4 cell line, Dia and de Mejia demonstrated that lunasin is able to internalize into the cell and sit within the nucleus, to modify the expression of human extracellular matrix and cell adhesion genes by binding to α_5_β_1_ integrin, and also to inhibit the FAK/ERK/NF-κB signaling pathway [16,18]. Indeed, peptides containing the RGD-motif can bind integrins and block their signaling pathways involved in cell adhesion, invasion and extracellular matrix components, mechanisms by which lunasin has recently shown inhibitory and anti-metastatic effects in some cancer models [15,50]. Studies have found that lunasin inhibits non small cell lung cancer cell proliferation acting as antagonist of αv integrin and histone acetylation modulatory agent [32,51]. Similarly, lunasin inhibited the migration and invasion properties of breast cancer cells via integrin-mediated FAK/Akt/ERK and nuclear factor (NF)-κβ pathways, and suppression of matrix metalloproteinases 2 and 9 [52]. The in vivo effect of this peptide was suggested in the CRC liver metastasis mice model by Dia and de Mejia [53], although disagreements between intraperitoneally- and orally-administered findings made it hard to establish a definitive lunasin´s role on preventing the CRC liver metastasis. Regarding the in vivo efficacy of lunasin against CSC, lunasin impaired the tumor growth initiated by CSC in a melanoma xenograft mouse model [35] and also suppressed the ability of these cancer-initiating cells to invade and proliferate in the lung of an experimental model of melanoma metastasis using B16-F10 cells [36].

## 3. Materials and Methods 

### 3.1. Materials

Peptide lunasin was synthesized by Chengdu KaiJie Biopharm Co., Ltd. (Chengdu, China). Its purity (>95%) was confirmed by liquid chromatography (HPLC) coupled to mass spectrometry (HPLC-MS).

### 3.2. Cell Lines

The human CRC cell line HCT-116 was obtained from American Type Cell Collection (ATCC, Manassas, VA, USA), and maintained in RPMI medium (ATCC) supplemented with 5% heat inactivated fetal bovine serum (FBS; Mediatech, Herndon, VA, USA), 100 units/mL penicillin, and 0.1 mg/mL streptomycin (Sigma-Aldrich, St. Louis, MO, USA). Cells were grown in a humidified incubator containing 5% CO_2_ and 95% air at 37 °C, kept sub-confluent, and medium was changed every other day. All cells were assayed within 5–25 passages. Enrichment culture of tumor-derived colon-spheres was performed by incubating parental HCT-116 cells in serum-free medium (SFM) composed of DMEM/F-12 medium supplemented with 2% B-27 supplement, 20 ng/mL recombinant human epidermal growth factor, 10 ng/mL fibroblast growth factor-basic (Life Technologies, Grand Island, NY, USA), 100 units/mL penicillin, 0.1 mg/mL streptomycin, and 10 μg/mL insulin (Sigma-Aldrich) in ultra low-attachment plates (Corning, Lowell, MA, USA) at 37 °C. Plated under these anchorage-independent conditions in supplemented-SFM, tumor cells form floating spheres reported to represent the growth of CSC [27,31,54]. 

### 3.3. Cell Proliferation Assay

HCT-116 cells were seeded in 96-well plates (1.1 × 10^4^ cells/mL). After 24 h incubation, cells were treated with different concentrations of lunasin ranging from 5 to 160 μM. After 72 h treatment, cell viability was determined by the MTT assay. Treatment medium was replaced by 200 μL of fresh medium containing 0.5 mg/mL MTT (Sigma-Aldrich). After 1 h incubation at 37 °C, MTT-containing medium was removed and the reduced formazan dye was solubilized by adding 100 μL of dimethyl sulfoxide to each well. After gently mixing, the absorbance was read at 570 nm using a microplate reader (Elx800TM, BioTek Instrument, Winooski, VT, USA). The results were expressed as percentage of the control, considered as 100%. Experiments were carried out in triplicate with at least three replicates per concentration.

### 3.4. Tumorsphere Formation Assay

To examine the effect of lunasin on the formation of tumorspheres derived from CRC HCT-116 cells, cells were grown in SFM and plated as single cells in ultra low-attachment 24-well plates (6 × 10^3^ cells/mL). Right after seeding, cells were treated with different concentrations of lunasin ranging from 5 to 160 μM and incubated at 37 °C for 10 days. After that time, tumorspheres were formed and transferred to 6-well dishes in differentiating medium (RPMI supplemented with 5% FBS and 1% antibiotics). Under these conditions, tumorspheres were adhered after 24 h incubation. Then, cells were stained with crystal violet solution (0.2% crystal violet in 2% ethanol) for 20 min at room temperature, photographed and counted. Results were presented as percentage of tumorspheres forming cells compared to control, considered as 100%. Analyses were performed in triplicate with at least three replicates per concentration.

### 3.5. Detection of Apoptosis

Apoptotic cells were quantified by Annexin V/PI double staining using an apoptotic detection kit (BioVision, Mountain View, CA, USA) according to manufacturer’s instruction, followed by flow cytometry. HCT-116 cells (4 × 10^4^ cells/mL) and colon tumorspheres (3 × 10^3^ cells/mL) were seeded onto 6-well plates and treated (20–80 μM lunasin) as described above. After 72 h treatment, HCT-116 cells were collected as described by Qiu and others [55]. In the case of colon tumorspheres, after 7 days treatment, floating cells in medium were collected in ice-cold flow cytometry tubes. After centrifugation (2000 × *g*, 2 min), single-cell suspensions were generated by incubation with 0.5 mL trypsin (0.25% trypsin-ethylenediaminetetraacetic acid, EDTA, Sigma-Aldrich) and 1 mL medium for 5 min at 37 °C, and gentle pippeting. Afterwards, in both cell cultures, cell suspensions were centrifuged (2000 × *g*, 2 min) and washed twice with 0.5 mL ice-cold phosphate buffer saline (PBS). Then, cells were suspended in 0.3 mL binding buffer containing Annexin V and PI, and incubated for 15 min at room temperature in the dark. Total apoptotic cells were identified using a BD LSR II cell analyzer (BD Biosciences, San Jose, CA, USA) as Annexin V-positive cells (apoptosis state), being further identified based on PI staining as early apopototic cells (Annexin V-positive/PI-negative) or late apoptotic-necrotic cells (Annexin V-positive/PI-positive). At least 10,000 events were recorded to assess the percentage of apoptotic cells. Analyses were performed in duplicate with at least three replicates per concentration, and results were presented as the increased number in apoptotic cell populations, compared to control cells.

### 3.6. Cell Cycle Analyses

HCT-116 cells and colon tumorspheres were treated as described for the apoptosis detection assay. After 72 h treatment, HCT-116 cells were collected as described by Qiu and others [55]. In the case of colon tumorspheres, after 7 days treatment, cells were collected as described for apoptosis detection assay with some modifications. Briefly, floating tumorspheres in medium were collected, centrifuged, and single-cell suspensions were generated, washed with ice-cold PBS, and then fixed in 1 mL of 70% ethanol and kept at −20 °C overnight. After centrifugation (2000× *g*, 2 min), cells were washed with 0.5 mL PBS, and incubated with 0.3 mL PBS solution containing RNAse (10%; Sigma-Aldrich) and PI (1%; BioVision) for 25 min at room temperature in the dark. Cell cycle distribution was analyzed with at least 8000 events recorded using a BD LSR II cell analyzer (BD Biosciences), and data were processed using ModFit LT software. Analyses were performed in duplicate with at least three replicates per concentration, and results were presented as percentage of cells in G1, S, and G2-phases.

### 3.7. Immunoblotting 

HCT-116 cells (3.5 × 10^4^ cells/mL) were seeded in 10 cm cell culture dishes. Colon tumorspheres were seeded exactly same as described for apoptosis assay. After 72 h treatment (20–80 μM lunasin), HCT-116 cells were collected and whole-cell lysates were prepared as previously described [53]. In the case of colon tumorspheres, after 7 days-treatment (20–80 μM lunasin), cells were collected following the same procedure with some modifications. Briefly, floating tumorspheres in medium were collected, centrifuged, and washed with ice-cold PBS. Then, cells were incubated on ice for 30 min in RIPA lysis buffer containing a protease inhibitor cocktail (Boston BioProducts, Ashland, MA, USA), and processed as previously described [56]. Supernatants were collected and protein content was quantified by the bicinchoninic acid method (Pierce, Rockford, IL, USA), using bovine serum albumin as standard protein. Equal amount of proteins (50–70 μg) were resolved over 12% SDS-polyacrylamide gel electrophoresis and transferred to nitrocellulose membranes. After blocking, membranes were incubated with different monoclonal primary antibodies overnight at 4 °C, according to manufacturer’s instructions. Primary antibodies for cleaved caspase-3 (Asp175), full-length PARP, p21^Waf1/Cip1^, and p27^Kip1^ were from Cell Signaling Technology (Beverly, MA, USA). β-actin was used as a loading control of cytosolic fraction, and its antibody was from Sigma-Aldrich. After 1 h incubation with the appropriate secondary antibodies (goat anti-mouse IgG, and goat anti-rabbit IgG IRDye (LI-COR Biosciences, Lincoln, NE, USA)), proteins of interest were visualized using enhanced chemiluminescence (Boston Bioproducts), processed with Image J Software and analyzed as we previously described [40]. 

### 3.8. Statistical Analysis

Data were evaluated using one-way ANOVA followed by Bonferroni post hoc test and expressed as the mean ± standard variation (SD) of the different experiments carried out. GraphPad Prism 5.0 software (San Diego, CA, USA) was used to perform statistical analyses. Differences with a *p* value < 0.05 (*), *p* value < 0.01 (**) or *p* value < 0.001 (***) were considered significant.

## 4. Conclusions

In the present study, our cellular model allowed us to approach the study of peptide lunasin towards the ideal evaluation of cancer-preventive agents by targeting both the parental and the stem-like tumorigenic populations. The protective mechanisms on lunasin-treated cells can be postulated in terms of inhibition of cell growth and tumorsphere-forming activity, induction of apoptosis, and regulation of cell cycle progression. The recent CSC hypothesis has supposed a challenge on the search of chemotherapeutic agents that efficiently target fast diving cancer cells as well as CSC responsible for the growth and maintenance of the tumorigenic bulk mass. To the best of our knowledge, this is the first study that suggests a protective role of lunasin against the formation of colon-spheres derived from CRC cells, specifically the HCT-116 cell line. The potential of bioactive peptides against the CSC subpopulation deserves additional studies characterizing CSC markers in more cellular models. Before concluding on lunasin´s effects over CSC, the promising results of this work clearly need to be further addressed to elucidate the molecular basis of the tumorsphere-inhibitory activity, to study its potential on stem-related markers and signaling pathways, such as Wnt/β-catenin, Hedgehog and Notch, and to confirm this role by using in vivo models of CSC self-renewal.

## Abbreviations

ATCC	American Type Cell Collection
CDK	Cyclin-dependent kinase
CRC	Colorectal cancer
CSC	Cancer stem-like cells
DHA	Docosahexaenoic acid
EDTA	Ethylenediaminitetraacetic acid
FBS	Fetal bovine serum
MTT	3-(4,5-dimethylthiazol-2-yl)-2,5-diphenyl tetrazolium bromide
PBS	Phosphate buffer saline
PI	Propidium iodide
SFM	Serum-free medium
